# Escitalopram or Novel Herbal Mixture Treatments during or following Exposure to Stress Reduce Anxiety-Like Behavior through Corticosterone and BDNF Modifications

**DOI:** 10.1371/journal.pone.0091455

**Published:** 2014-04-01

**Authors:** Ravid Doron, Dafna Lotan, Ziv Versano, Layla Benatav, Motty Franko, Shir Armoza, Nadav Kately, Moshe Rehavi

**Affiliations:** 1 School of Behavioral Sciences, Academic College of Tel Aviv-Yaffo, Tel- Aviv, Israel; 2 Department of Education and Psychology, The Open University of Israel, Raanana, Israel; 3 School of Health and Life Sciences, Hadassah Academic College, Jerusalem, Israel; 4 School of Psychological Sciences and Sagol School of Neuroscience, Tel-Aviv University, Tel- Aviv, Israel; 5 Broshim Campus, Tel-Aviv University, Tel-Aviv, Israel; 6 Department of Physiology and Pharmacology, Sackler Faculty of Medicine, Tel-Aviv University, Tel-Aviv, Israel; 7 The Dr. Miriam and Sheldon G. Adelson Chair in the Biology of Addictive Diseases, Sackler Faculty of Medicine, Tel-Aviv University, Tel-Aviv, Israel; Radboud University, Netherlands

## Abstract

Anxiety disorders are a major public health concern worldwide. Studies indicate that repeated exposure to adverse experiences early in life can lead to anxiety disorders in adulthood. Current treatments for anxiety disorders are characterized by a low success rate and are associated with a wide variety of side effects. The aim of the present study was to evaluate the anxiolytic effects of a novel herbal treatment, in comparison to treatment with the selective serotonin reuptake inhibitor escitalopram. We recently demonstrated the anxiolytic effects of these treatments in BALB mice previously exposed to one week of stress. In the present study, ICR mice were exposed to post natal maternal separation and to 4 weeks of unpredictable chronic mild stress in adolescence, and treated during or following exposure to stress with the novel herbal treatment or with escitalopram. Anxiety-like behavior was evaluated in the elevated plus maze. Blood corticosterone levels were evaluated using radioimmunoassay. Brain derived neurotrophic factor levels in the hippocampus were evaluated using enzyme-linked immunosorbent assay. We found that (1) exposure to stress in childhood and adolescence increased anxiety-like behavior in adulthood; (2) the herbal treatment reduced anxiety-like behavior, both when treated during or following exposure to stress; (3) blood corticosterone levels were reduced following treatment with the herbal treatment or escitalopram, when treated during or following exposure to stress; (4) brain derived neurotrophic factor levels in the hippocampus of mice treated with the herbal treatment or escitalopram were increased, when treated either during or following exposure to stress. This study expands our previous findings and further points to the proposed herbal compound's potential to be highly efficacious in treating anxiety disorders in humans.

## Introduction

Stress and anxiety disorders are a major public health concern worldwide. They are recognized as a main risk factor for many diseases, including cardiovascular, metabolic and neuropsychiatric disorders [1], [2]. Epidemiologic studies indicate that repeated exposure to adverse experiences early in life is associated with increased risk for developing anxiety disorders, probably due to neurobiological changes as well as alterations in the hypothalamic-pituitary-adrenal (HPA) axis that follow these experiences [3].

Finding suitable treatments for anxiety disorders is of the utmost importance. Current treatments include benzodiazepines and selective serotonin reuptake inhibitors (SSRIs). Benzodiazepines can only be prescribed for short periods to avoid tolerance and physical dependence [4]. By definition, however, the chronic nature of anxiety disorders requires long-term treatment [5]. SSRIs are prescribed for a long term treatment of anxiety disorders, and have been shown to be effective in treating a wide spectrum of these disorders [4]. Nevertheless, they are characterized by a low success rate and are associated with a variety of side effects [6], [7].

These disadvantages have driven the research of alternative treatments. Studies have shown therapeutic potential of chronic treatment with several herbal medicines, such as St. John's Wort [8], [9] and Kava [9], [10], in various psychiatric disorders. Chronic treatment with several herbal medicines have been shown to normalize stress hormone levels [11], as well as brain neurotransmitter levels [12], [13], in a similar manner to the influences of SSRIs treatment, in both in-vitro and in-vivo animal studies. Therefore, herbal medicines might serve as an efficient treatment for anxiety disorders.

The aim of the present study was to evaluate the behavioral and biological anxiolytic effects of a novel herbal treatment (NHT), in comparison to conventional treatment with escitalopram. The NHT was prepared from the following four components: Crataegus Pinnatifida, Triticum Aestivu, Lilium Brownie and Fructus Zizyphi Jujubae, as a modification of a classical Chinese formula used in the treatment of mental disorders since the 2^nd^ century A.D [14]. We have recently found that treatment of BALB mice previously exposed to stress with the NHT has reduced anxiety-like behavior, normalized the stress response, and increased BDNF levels in the hippocampus [15]. These effects were similar to the ones found following treatment with the SSRI escitalopram. In the present study, we further evaluated the behavioral and biological anxiolytic effects of the NHT. Both the infant and the adolescence periods are specific windows of vulnerability, in which exposure to stress can impact the normal development of the HPA axis [16]–[18]. Thus, mice were exposed to post-natal maternal separation (MS) and to unpredictable chronic mild stress (UCMS) in adolescence, and their anxiety-like behavior was evaluated in adulthood. The anxiolytic effects were examined on mice which underwent MS and UCMS, and treated with NHT either *during* or *following* exposure to stress, in comparison to treatment with the SSRI escitalopram. Finally, blood corticosterone and brain BDNF levels were evaluated in these mice.

## Materials and Methods

### Ethics statement

All experiments were approved by the Institutional Animal Care and Use Committee of the Academic College of Tel Aviv-Yaffo (mta-2012-16-4). All efforts were made to minimize animal suffering.

### Animals

For all experiments, ICR outbred mice (Harlan, Israel) were bred and kept in the vivarium of the ‘Academic College of Tel Aviv-Yafo'. Mice were housed in standard group cages (5 mice per cage, each cage contains mice from all experimental groups), kept on a reversed 12 h light/dark cycle (lights on 1900–0700) and given ad libitum access to food and water. All experiments were performed during the dark phase under red light. The animals receiving the NHT did not experience any physical or behavioral adverse effects.

### Drugs

Crataegus Pinnatifida, Triticum Aestivu, Lilium Brownie and Fructus Zizyphi Jujubae were purchased as freeze-dried granules from KPC Products, Inc (Irvine, CA, USA). Escitalopram was kindly donated by TEVA Ltd (Israel). NHT was prepared by dissolving the 4 compounds (together) in saline containing 1% DMSO to give a final concentration of 0.47 mg/ml (each). The NHT was administered daily at a dose of 15 or 30 mg/kg (i.p injection). Escitalopram was administered daily at a dose of 15 mg/kg (i.p injection). The dose chosen for escitalopram was based on previous studies [19], [20]. The same dose and a double dose were chosen for the NHT treatment.

### Biochemical assessment

#### Assessment of corticosterone levels

Blood was collected following 3 weeks of treatment and behavioral assessment, from the facial vein (approx. 0.1 ml) into EDTA-coated tubes. All blood samples were kept on ice and plasma was collected after centrifugation for 10 min (2200 g, 4°C). Plasma samples were stored at −20°C until the determination of corticosterone levels by RIA (^125^I RIA kits, MP Biomedicals, Orangeburg, NY), as per the manufacturers' instructions.

#### Assessment of brain BDNF levels

Tissue samples were obtained as previously described [21]. Shortly, mice were decapitated, and their brains were placed on ice. Serial sections were cut onto slides and tissue punches of the hippocampus and prefrontal cortex (PFC) were taken. Tissue punches were homogenized in cold extraction buffer (Tris-buffered saline, pH 8.0, with 1% NP-40, 10% glycerol, 5 mM sodium metavanadate, 10 mM PMSF, 100 μg/ml aprotinin and 10 μg/ml leupeptin). Homogenates were acidified with 0.1 M HCl (pH 3.0), incubated at room temperature (22–24°C) for 15 min, and neutralized with 0.1 M NaOH (pH 7.6). Homogenates were then microfuged at 7,000 g for 10 min. BDNF levels were evaluated using sandwich enzyme-linked immunosorbent assay as previously described [22]. BDNF concentrations are presented after normalization to total protein levels.

### Stress procedures - Behavioral/Psychological stress

#### Maternal Separation (MS)

On the day of parturition (postnatal day, PND 0), pups underwent a MS procedure. Once per day, from PND 0 to PND 14 pups were removed from their home cage and placed together in a separate room and in a separate clean cage, situated on a heating pad (30–33°C) in order to keep the pups in a temperature approximating the dam's external body temperature. Six hours later, pups were returned to their home cage.

#### Unpredictable chronic mild stress (UCMS)

This paradigm is based on the procedure originally designed by Willner [23] et al. and adapted to mice [24]. The procedure was performed during adolescence, starting at the age of 4 weeks. Treated mice were subjected to UCMS for 4 weeks, using the following stressors: placement in an empty cage with 1 cm of water at the bottom (water stress), inducing social stress by placing mice in soiled cages of other mice, inversing the light/dark cycle, placing mice in cages with wet sawdust, tilt cages at 30 degrees and restraining the mice. To prevent habituation and to provide an unpredictable feature to the stressors, stressors were administered at different time points during the day (see Table 1).

**Table 1:** Unpredictable chronic mild stress (UCMS) procedure.

	Day 1	Day 2	Day 3	Day 4	Day 5	Day 6	Day 7
**Week 1**	Restraint stress	Placement in an empty cage with water at the bottom + Lights on	Tilt cages at 30 degrees	Placing mice in cages with wet sawdust	Placing mice in soiled cages of other mice	Restraint stress	Reversal of the light/dark cycle
	(4 hours)	(4 hours)	(4 hours)	(4 hours)	(4 hours)	(4 hours)	
**Week 2**	Placement in an empty cage with water at the bottom + Lights on	Tilt cages at 30 degrees	Placing mice in cages with wet sawdust	Placing mice in soiled cages of other mice	Restraint stress	Placement in an empty cage with water at the bottom + Lights on	Reversal of the light/dark cycle
	(4 hours)	(4 hours)	(4 hours)	(4 hours)	(4 hours)	(4 hours)	
**Week 3**	Tilt cages at 30 degrees	Placing mice in cages with wet sawdust	Placing mice in soiled cages of other mice	Restraint stress	Placement in an empty cage with water at the bottom + Lights on	Tilt cages at 30 degrees	Reversal of the light/dark cycle
	(4 hours)	(4 hours)	(4 hours)	(4 hours)	(4 hours)	(4 hours)	
**Week 4**	Placing mice in cages with wet sawdust	Placing mice in soiled cages of other mice	Restraint stress	Placement in an empty cage with water at the bottom + Lights on	Tilt cages at 30 degrees	Placing mice in cages with wet sawdust	Reversal of the light/dark cycle
	(4 hours)	(4 hours)	(4 hours)	(4 hours)	(4 hours)	(4 hours)	

Treated mice were subjected to UCMS for 4 weeks, using the listed stressors. To prevent habituation and to provide an unpredictable feature to the stressors, stressors were administered at different time points during the day.

### Behavioral assessment

#### Elevated plus-maze (EPM)

This task is based on the natural tendency of mice to avoid open and elevated places [25]. The apparatus, situated 40 cm above the floor, consists of a plus-maze with two black plastic closed arms and two opposite open arms. Each arm is 64 cm long and 5 cm wide. Each mouse was placed in the center of the EPM and its behavior video recorded for 5 min, and later coded by an observer blind to the mouse treatment, using the Biobserve software. The maze was thoroughly cleaned with ethanol and allowed to dry between subjects in order to eliminate any odor cues. Anxiety-like behavior was measured by the time the animals spent in the open, unprotected arm of the maze. The number of closed arm entries was used to assess motor activity [26].

### Experimental design

#### Experiment 1: Evaluating the effects of exposure to stress early in life on anxiety-like behavior

Due to a discrepancy of results in animal studies using different models to induce anxiety-like behavior, we evaluated the impact of exposure to stress in two life periods: neonatal and adolescence, on anxiety-like behavior in adulthood. On the day of parturition, pups were randomly assigned to undergo chronic stress during the postnatal (MS) and adolescence (UCMS) periods. Upon reaching adulthood both groups were tested for anxiety-like behavior using the EPM.

#### Experiment 2: Evaluating the anxiolytic effects of NHT following exposure to stress early in life

**Figure 1:**
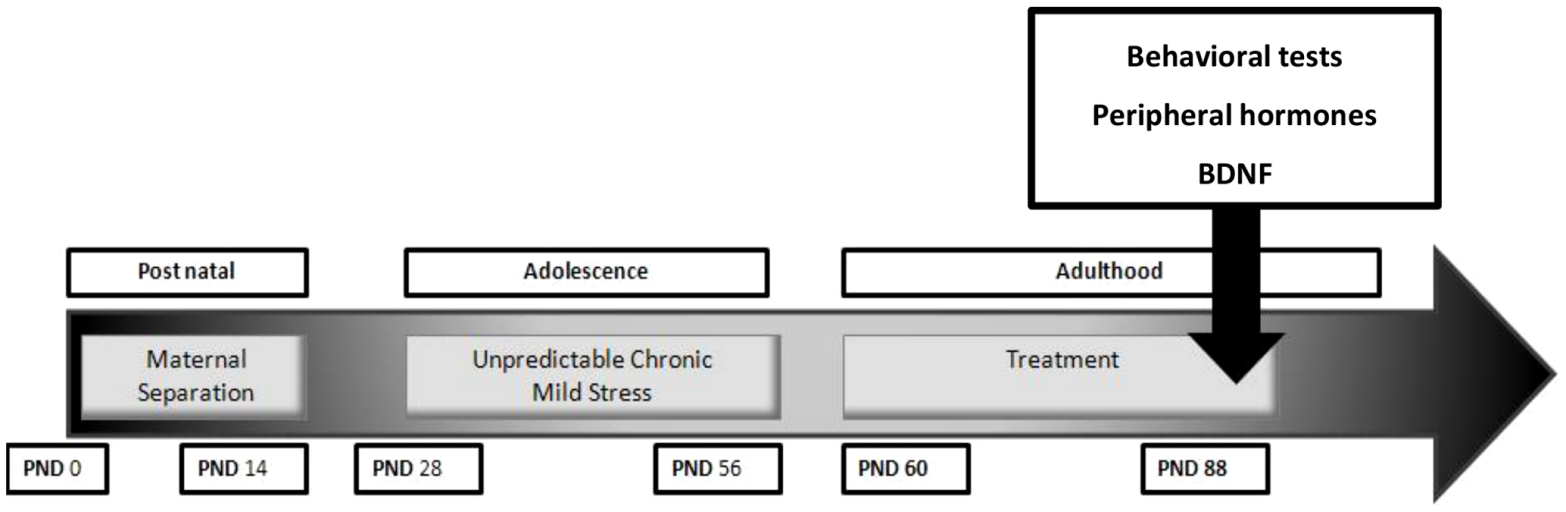
Experiment 2 outline.

This experiment evaluated the behavioral and physiological effects of NHT *following* exposure to stress. On the day of parturition, pups were exposed to chronic stress during the postnatal (MS) and adolescence (UCMS) periods. Upon reaching adulthood, these mice were randomly assigned into one of 4 treatment groups: (a and b) NHT at the dose of 15 mg/kg or 30 mg/kg (respectively), (c) escitalopram (15 mg/kg), (d) a control group receiving only the vehicle. Following a 3-week administration of the relevant drug or vehicle, anxiety-like behavior, peripheral corticosterone levels and hippocampus BDNF levels were evaluated (for time line, see Fig. 1).

#### Experiment 3: Evaluating the anxiolytic effects of NHT during adverse experiences

**Figure 2:**
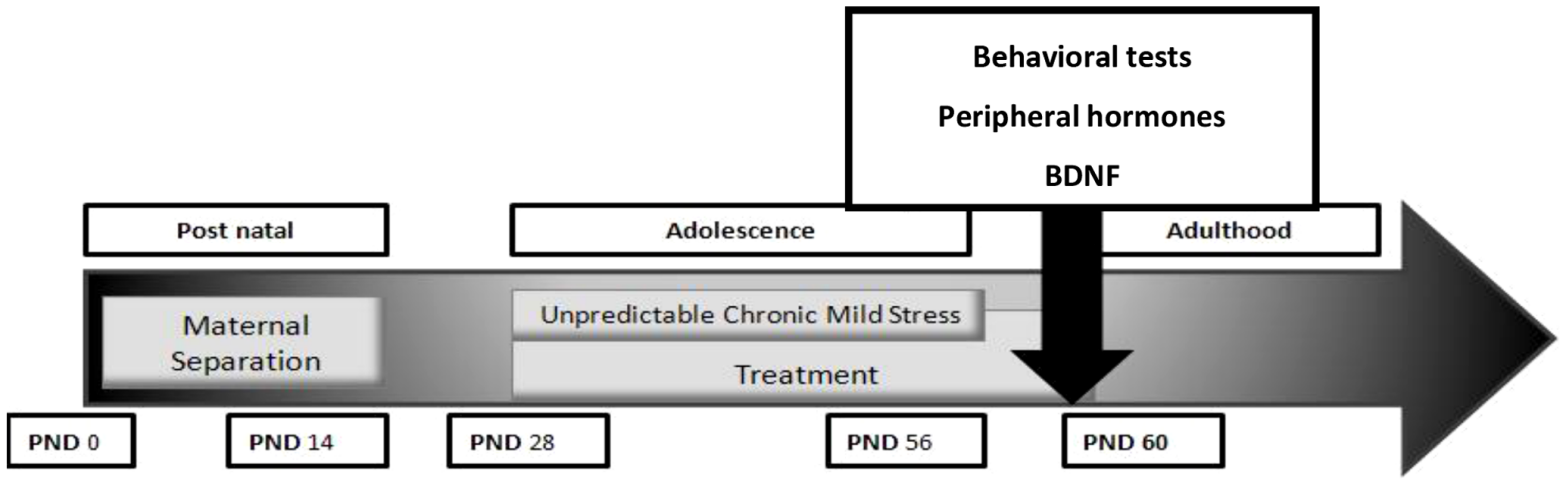
Experiment 3 outline.

The third experiment examined the ability of NHT to prevent lasting effects of anxiety and to evaluate the protective properties of NHT when treated *during* exposure to stress in adolescence. Therefore, mice previously exposed to post-natal stress (MS) were treated while undergoing stress in adolescence (UCMS). At the age of 28 days pups were randomly assigned into one of 4 groups of treatment: (a and b) the NHT at the dose of 15 mg/kg or 30 mg/kg, (c) escitalopram (15 mg/kg), (d) a control group which underwent the two stress procedures (MS+UCMS) and received only the vehicle. Upon reaching adulthood, anxiety-like behavior, peripheral corticosterone levels and hippocampus BDNF levels were assessed (for time line, see Fig. 2).

### Data analysis and interpretation of results

Results are expressed as mean ± SEM. Data was analyzed using: (a) two-sample Student's t-tests to compare means of time spent in the open arms of the EPM between chronic stressed and non-stressed mice; (b) one-way ANOVA with Treatment as a between subject variable, performed on corticosterone blood levels; hippocampus BDNF levels; the time spent in the open arms of the EPM; and the number of closed arms entries. Where results were significant, the ANOVA was followed by a planned contrasts analysis. Significance was assumed at p<0.05.

## Results

### Experiment 1: Exposure to stress early in life led to increased anxiety-like behavior in adulthood

**Figure 3:**
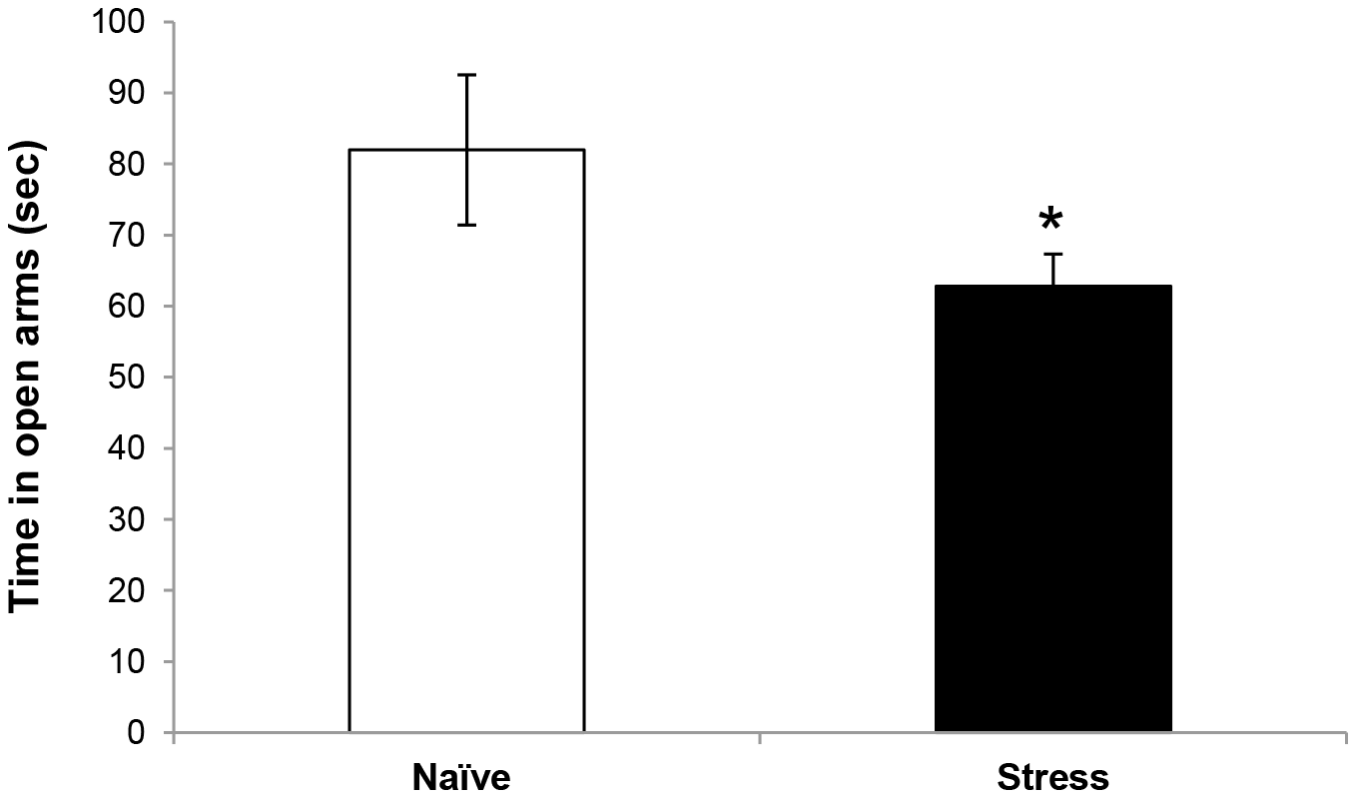
Effect of stress manipulation on anxiety-like-behavior in the Elevated Plus Maze. The time spent in open arms of the EPM was significantly higher in control mice (n = 17), compared to stressed mice (n = 51). Significantly different from the control group: *p<0.05.

Figure 3 presents anxiety-like behavior *following* exposure to stress early in life. Mice exposed to MS and UCMS displayed increased anxiety-like behavior, as expressed in a significant reduction in the time spent in the open arms of the EPM, in comparison to control mice (Fig. 3, stressed group: 62.8±4.5 sec, n = 51; naïve group: 82.0±10.6 sec, n = 17; t(66) = 1.942, p<0.05).

### Experiment 2: Treatment with NHT *following* exposure to stress early in life had behavioral and biological anxiolytic effects

**Figure 4:**
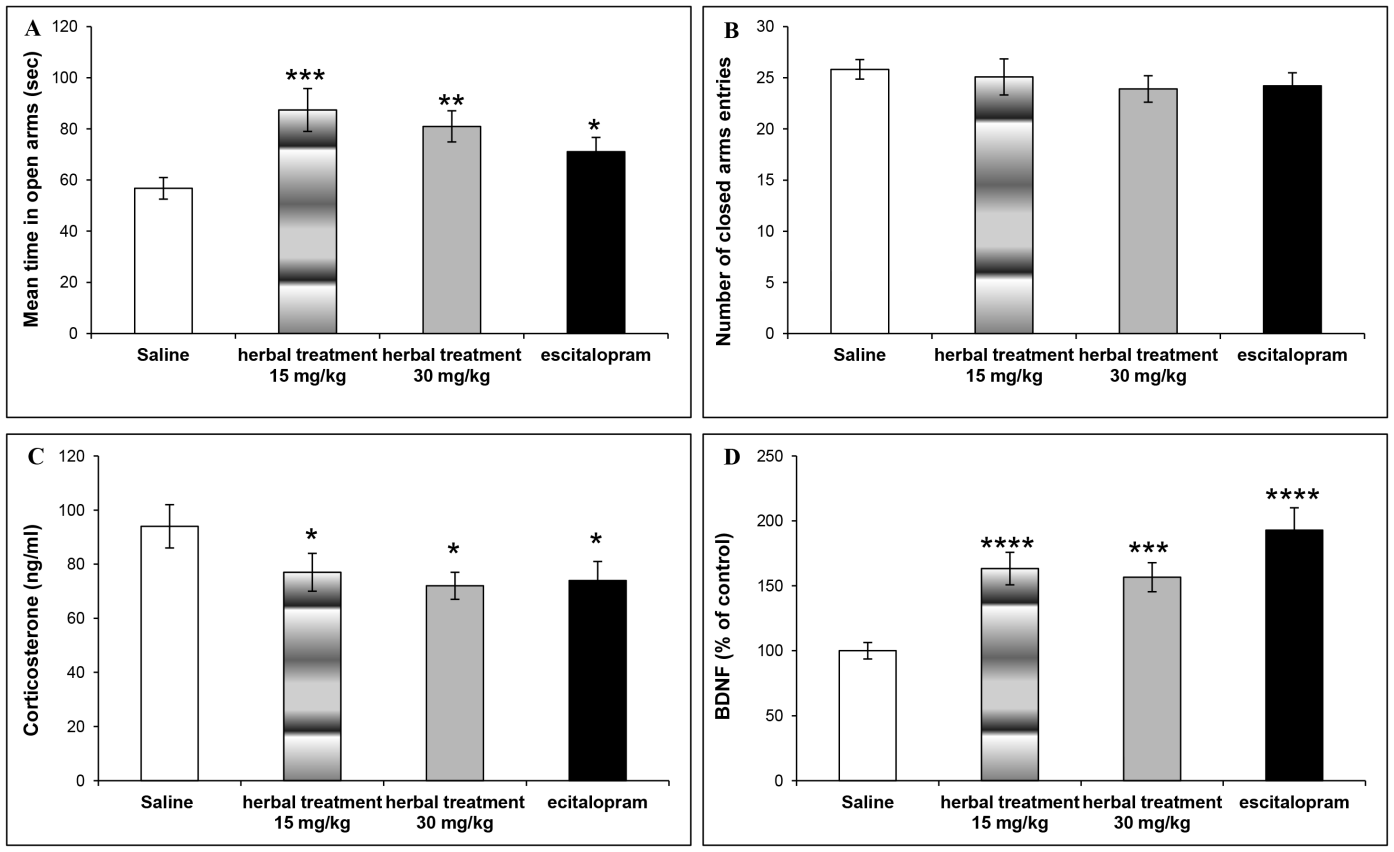
Behavioral and biological effects of NHT and escitalopram treatments *following* exposure to stress. Figure 4A: Treatment effect on time spent in the open arms of the EPM (saline: n = 40; NHT 15 mg/kg: n = 24; NHT 30 mg/kg: n = 22; escitalopram: n = 23). Figure 4B: Treatment effect on number of EPM closed arms entries (saline: n = 40; NHT 15 mg/kg: n = 24; NHT 30 mg/kg: n = 22; escitalopram: n = 23). Figure 4C: Treatment effect on blood corticosterone levels (saline: n = 17; NHT 15 mg/kg: n = 18; NHT 30 mg/kg: n = 18; escitalopram: n = 16). Figure 4D: Treatment effect on hippocampus BDNF levels (saline: n = 26; NHT 15 mg/kg: n = 24; NHT 30 mg/kg: n = 22; escitalopram: n = 23). Significantly different from the control group: *p<0.05, **p<0.005, ***p<0.0005, ***p<0.0001.

Figure 4 presents time in the open arms of the EPM (Fig. 4A), number of closed arms entry (Fig. 4B), corticosterone blood levels (Fig. 4C), and BDNF levels in the hippocampus (Fig. 4D) of mice treated following exposure to stress. Treatment with NHT reduced anxiety-like behavior in the EPM, compared to control mice, as expressed in a significant reduction in the time spent in the open arms of the EPM (Fig. 4A, saline: 56.8±4.2 sec, n = 40; NHT 15 mg/kg: 87.4±8.4 sec, n = 24; NHT 30 mg/kg: 81.0±6.1 sec, n = 22; escitalopram: 71.0±5.6 sec, n = 23; one way ANOVA: F(3,105) = 5.864, p<0.001). Planned comparisons between control and treatment groups demonstrated a reduction in time spent in the open arms of the EPM in the two NHT groups (15 and 30 mg/kg per day, p<0.0001 and p<0.005, respectively) and the escitalopram group (p<0.05). There were no differences in motor activity between the different treatment groups and the control group (Fig. 4B, saline: 25.8±1.0 entries, n = 40; NHT 15 mg/kg: 25.1±1.0 entries, n = 24; NHT 30 mg/kg: 23.9±1.3 entries, n = 22; escitalopram: 24.3±1.3 entries, n = 23; one way ANOVA: F(3,105) = 0.497, p = 0.685).

On the biological level, treatment has significantly reduced corticosterone blood levels (Fig. 4C, saline: 94.0±8.3 ng/ml, n = 17; NHT 15 mg/kg: 76.6±7.1 ng/ml, n = 18; NHT 30 mg/kg: 71.8±5.3 ng/ml, n = 18; escitalopram: 74.4±6.7 ng/ml, n = 16; one way ANOVA: F(3,65) = 2.102, p = 0.0545). Planned comparisons between control and treatment groups demonstrated a reduction in corticosterone blood levels in the NHT (15 and 30 mg/kg per day) and escitalopram (15 mg/kg per day) groups (p<0.05 for all comparisons). In addition, treatment has significantly increased BDNF levels in the hippocampus relative to control (Fig. 4D, saline: 100±6.3%, n = 26; NHT 15 mg/kg: 163.3±12.5%, n = 24; NHT 30 mg/kg: 156.6±11.2%, n = 22; escitalopram: 192.9±17.2%, n = 23; one way ANOVA: F(3,91) = 10.648, p<0.0001). Planned comparisons between control and treatment groups demonstrated an increase in BDNF in all treatment groups (15 mg/kg per day and escitalopram, p<0.0001; 30 mg/kg per day, p<0.0005).

### Experiment 3: The NHT prevented anxiety-like behavior and motor alterations when treated *during* exposure to stress in adolescence

**Figure 5:**
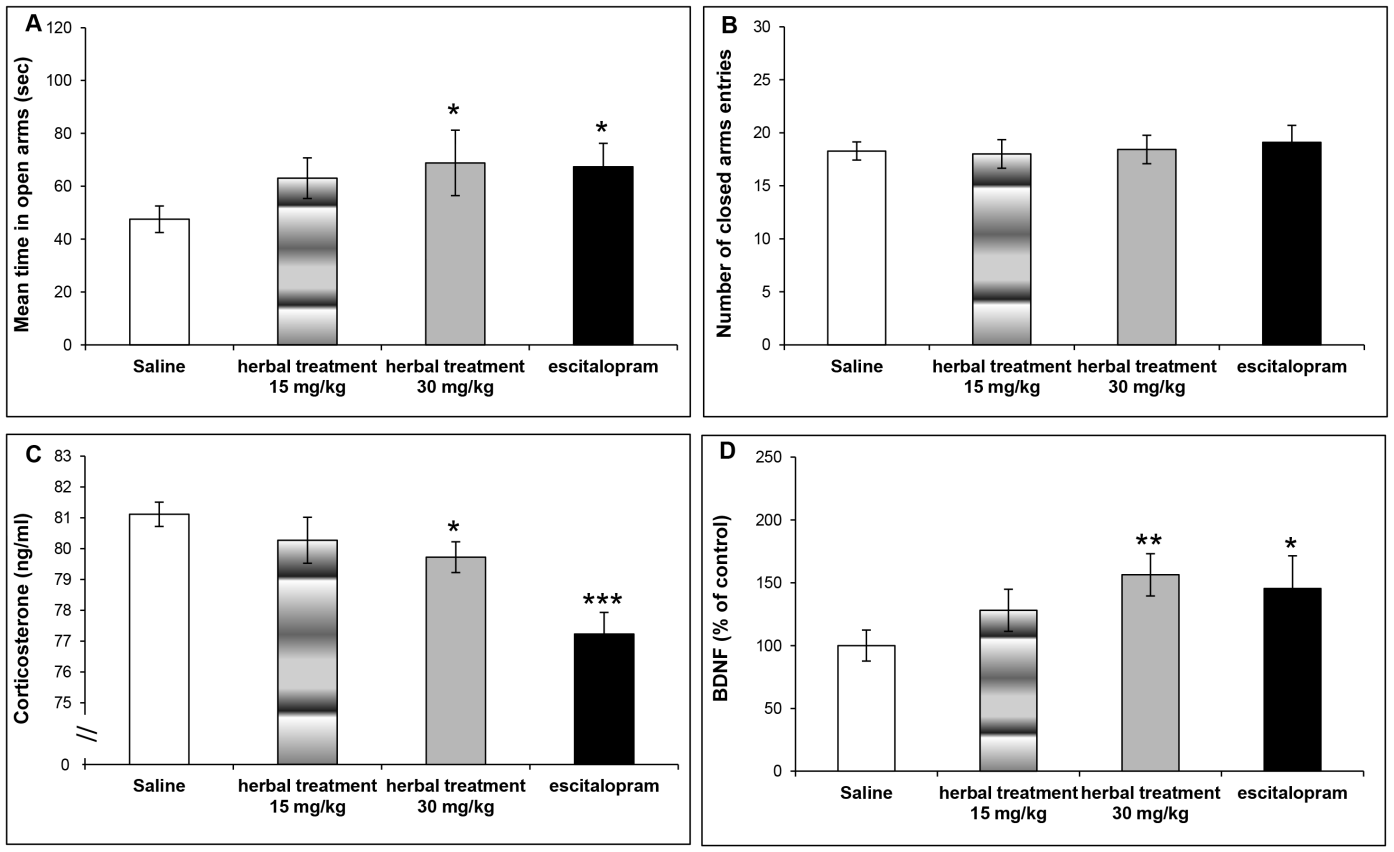
Behavioral and biological effects of the NHT and escitalopram treatment *during* exposure to stress. Figure 5A: Treatment effect on time spent in the open arms of the EPM (saline: n = 25; NHT 15 mg/kg: n = 20; NHT 30 mg/kg: n = 19; escitalopram: n = 20). Figure 5B: Treatment effect on number of EPM closed arms entries (saline: n = 25; NHT 15 mg/kg: n = 20; NHT 30 mg/kg: n = 19; escitalopram: n = 20). Figure 5C: Treatment effect on blood corticosterone levels (saline: n = 24; NHT 15 mg/kg: n = 17; NHT 30 mg/kg: n = 17; escitalopram: n = 17). Figure 5D: Treatment effect on hippocampus BDNF levels (saline: n = 20; NHT 15 mg/kg: n = 15; NHT 30 mg/kg: n = 12; escitalopram: n = 10). Significantly different from the control group: *p<0.05, **p<0.01, ***p<0.0001.

Figure 5 presents time in the open arms of the EPM (Fig. 5A), number of closed arms entry (Fig. 5B), corticosterone blood levels (Fig. 5C), and BDNF levels in the hippocampus (Figure 5D) of mice treated *during* exposure to stress. Treatment with either NHT (30 mg/kg per day) or escitalopram (15 mg/kg per day) reduced anxiety-like behavior in the EPM, compared to control mice, as expressed in a significant reduction in the time spent in the open arms of the EPM (Fig. 5A, saline: 47.5±5.0 sec, n = 25; NHT 15 mg/kg: 63.1±77 sec, n = 20; NHT 30 mg/kg: 68.8±12.4 sec, n = 19; escitalopram: 67.4±8.8 sec, n = 20; one way ANOVA: F(3,80) = 1.474, p = 0.228). Planned comparisons between control and treatment groups demonstrated a reduction in time spent in the open arms of the EPM in the NHT (30 mg/kg per day) and escitalopram (15 mg/kg per day) groups (p<0.05). There were no differences in motor activity between the different treatment groups and the control group (Fig. 5B, saline: 18.3±0.9 entries, n = 25; NHT 15 mg/kg: 18.0±1.4 entries, n = 20; NHT 30 mg/kg: 18.4±1.34 entries, n = 19; escitalopram: 19.1±1.6 entries, n = 20; one way ANOVA: F(3,80) = 0.13, p = 0.942).

Treatment has significantly reduced corticosterone blood levels (Fig. 5C, saline: 81.0±0.4 ng/ml, n = 24; NHT 15 mg/kg: 80.3±0.8 ng/ml, n = 17; NHT 30 mg/kg: 79.7±0.5 ng/ml, n = 17; escitalopram: 77.2±0.7 ng/ml, n = 17; one way ANOVA: F(3,71) = 8.489, p<0.0001). Planned comparisons between control and treatment groups demonstrated a reduction in corticosterone blood levels in the NHT (30 mg/kg per day) and escitalopram (15 mg/kg per day) groups (p<0.05 and p<0.0001, respectively). Treatment with either NHT (30 mg/kg per day) or escitalopram (15 mg/kg per day) significantly increased BDNF levels in the hippocampus, relative to control (Fig. 5D, saline: 100±12.4%, n = 20; NHT 15 mg/kg: 128.1±16.8%, n = 15; NHT 30 mg/kg: 156.3±16.8%, n = 12; escitalopram: 145.5±25.9%, n = 10; one way ANOVA: F(3,53) = 2.332, p = 0.085). Planned comparisons between control and treatment groups demonstrated an increase in hippocampus BDNF levels in the NHT (30 mg/kg per day) and escitalopram (15 mg/kg per day) groups (p<0.01 and p<0.05, respectively).

## Discussion

The present study explored the anxiolytic effects of a NHT, in comparison with the conventional treatment with the SSRI escitalopram. Our study led to three main findings: (1) Exposure of mice to MS during the postnatal period and to UCMS in the adolescence period led to increased anxiety-like behavior in these mice, compared to naïve mice. (2) The NHT reduced anxiety-like behavior as well as blood corticosterone levels, and increased hippocampus BDNF levels following postnatal MS and adolescence UCMS procedures. (3) Treatment with NHT during adolescence UCMS exposure has reduced anxiety-like behavior and blood corticosterone levels, and increased hippocampus BDNF levels following postnatal MS.

In order to evaluate the anxiolytic effects of NHT, it was first required to induce anxiety-like behavior in mice. Epidemiologic studies indicate that exposure to stress during childhood or adolescence can increase the risk for developing anxiety disorders, probably due to neurobiological changes that follow these experiences [3]. The neonatal period is an important developmental period, in which exposure to various stressors can influence different brain structures as well as the normal HPA axis function. In humans, a childhood history of neglect or exposure to traumatic events are linked to the development of affective disorders [27]. Likewise, exposure of rodents to prolonged periods of MS during the neonatal period has led to enhanced stress responsiveness, long lasting neurochemical changes and anxiety-like behaviors in adulthood [16], [28]–[33]. Studies in human adolescents suggest that this period is associated with basal and stress-induced increased activity of the HPA axis [17], [18], which may be related to the major changes in sex steroid levels during this period. In adolescent rodents, HPA-axis function is characterized by a prolonged activation in response to stressors compared with adulthood after exposure to the same stressor [34]. These findings point at an interaction between the developmental stage and the animal HPA axis responses to stress and support our results, demonstrating increased anxiety-like behavior following exposure of mice to both the MS and UCMS. Therefore, in the following experiments we exposed mice to MS in the post-natal period and to UCMS in adolescence.

One of the most important findings of our study was that NHT reduced anxiety-like behavior as well as blood corticosterone levels, and increased BDNF levels in the hippocampus of mice that were previously exposed to stress (MS+UCMS). These anxiolytic effects were similar to the ones observed following escitalopram treatment. These findings are in accordance with our previous study [15], in which treatment with NHT or escitalopram have reduced anxiety-like behavior and corticosterone levels, and increased BDNF levels in the hippocampus of BALB mice, previously exposed to a one-week of stress [15]. SSRIs treatment is currently one of the main treatments for anxiety disorders. It has been repeatedly shown that SSRIs can reduce anxiety symptoms in patients suffering from anxiety disorders [4], [35], [36], and anxiety-like behaviors in animal studies [37], [38]. Part of the SSRIs anxiolytic effects might be a result of amelioration in the HPA axis function. Abnormalities in the HPA axis functioning have been repeatedly documented in patients suffering from anxiety disorders [39], [40]. SSRIs treatments have been shown to reduce cortisol levels in these patients [41], [42]. Similarly, animal studies have displayed a reduction in corticosterone levels following SSRIs treatment [43], [44]. Therefore, it is hypothesized that a normalization of the HPA axis might, at least in part, be involved in the mechanism by which SSRIs create their anxiolytic therapeutic effects. A reduction in corticosterone levels, both following our NHT and escitalopram treatment, might point at a similar mechanism by which the novel treatment and SSRIs yield their beneficial effects.

Another biological factor that might contribute to the beneficial effects of NHT is BDNF. It has been demonstrated that BDNF is involved both in the pathogenesis and treatment of anxiety disorders [45], [46]. In humans, polymorphism in the BDNF gene has been associated with anxiety-related behaviors and a reduction in the hippocampus volume, especially in individuals exposed to early life stress [47], [48]. Exposure to stress early in life can alter the normal neuronal development, thus causing long lasting brain function alterations. This can partly be attributed to a long-term reduction in BDNF levels in the brain following exposure to stress in this period [49], [50]. Chronic SSRI treatment leads to an increase in BDNF levels in the hippocampus, and its therapeutic effect is dependent on this increase. Therefore, our finding of increased BDNF levels in the hippocampus following both NHT and escitalopram strengthen the assumption of a common biological pathway through which the NHT and escitalopram yields their anxiolytic effects.

Our third goal was to study whether our NHT could prevent the pathophysiology of anxiety by applying the treatment during exposure to stress in the adolescence period. We found that NHT has indeed decreased anxiety-like behavior as well as corticosterone levels and increased hippocampus BDNF levels when treated during UCMS. Treatment with escitalopram has yield similar effects. Studies in humans and rodents have been repeatedly linked adolescent stress experience to permanent dysregulation of the HPA axis, which can increase the probability of developing anxiety disorders [51]. Exposure to stress in adolescents can alter normal brain development and plasticity, at least in part due to increased activity of the HPA axis and a decrease in BDNF hippocampus levels. It is noteworthy that the elevation in the HPA functioning following exposure to stress might be the cause of the reduction in BDNF levels in the brain [52], [53]. Treatment during the adolescence period probably prevented the HPA axis alterations caused by exposure to stress in this period, which in turn might prevent the reduction in brain BDNF levels and to developmental brain and behavioral abnormalities. Studies have shown that SSRI treatment during exposure to stress prevented the reduction in blood BDNF levels in humans [54] and in rodents [55] and the increase in corticosterone blood levels in mice [44]. These findings are extremely important, because they suggest the possibility of preventing anxiety disorders in children and adolescents that are exposed to a traumatic event- by treating them, if possible, during or immediately following this event.

This study expands our previous findings regarding the anxiolytic effects of the NHT, and further demonstrates the potential anxiolytic effects of a NHT, both by administration of the treatment following exposure to stress and as a prophylactic agent preventing the behavioral and biological alteration caused by exposure to stress early in life. Based on these two studies, our NHT has the potential to be highly efficacious in treating anxiety disorders and thus may prove to be an excellent candidate as a novel treatment for anxiety disorders in humans. The fact that NHT did not lead to sexual dysfunction [56], one of the main side effects of SSRIs treatment, can reduce the incidences of treatment discontinuation often observed in SSRI medicated patients. In addition, the NHT is prepared from food additives, which can both lessen patients worries regarding its consumption safety and will simplify the transition of the treatment from animal studies to clinical studies- and finally, to patients. Revealing the mechanisms that stand in the basis of this new treatment will not only further our understanding of its specific beneficial effects on behavior, but will help us further our understanding of the biological mechanisms underlying anxiety disorders, which are still poorly understood.
